# Recent Contributions to the Literature of Comparative Medicine

**Published:** 1880-10

**Authors:** Thomas E. Satterthwaite


THE ARCHIVES

OF

### COMPARATIVE MEDICINE AND SURGERY.

VOL. 1. OCTOBER, 1880. No. 4.

### Original Articles, Selections, and Translations.

### ART. XVII.—SOME OF THE RECENT CONTRIBUTIONS TO THE LITERATURE OF COMPARATIVE MEDICINE.

### RABIES IN THE LOWER ANIMALS.—THE ECCHINOCOCCUS AND CYSTICERCUS CELLULOSÆ DISEASES.

BY THOMAS E. SATTERTHWAITE, M. D.

PART I.

RABIES IN THE LOWER ANIMALS.—There are quite a number of diseases which are communicable from the lower animals to man or conversely. The clinical history of each, altered as it is by the differing conditions, presents an abundance of food for study, and has great practical value, both for the veterinarian and the practitioner of human medicine. Upon these topics, indeed, the one physician ministers to the other, while the knowledge which each has gained is essential to furnish a complete picture of the diseases in all their details.

The following notes have, therefore, a double interest in so far as they belong to the border-land just alluded to—a ground which is particularly important just now, to all students of general pathology. An interesting consideration that attracts the attention of one who is reviewing the literature of hydrophobia, is the utter impropriety of its name, whether used to indicate the disease of men or animals. If intended to imply a symptom in the non-human form, the inference is erroneous, for the affected animals have no dread of water. Nor is hydrophobia, as a symptom, pathognomonic of the human disease, since it has been observed in various morbid states of a nervous character. In one instance, given by Mr. Dolan, where there was plain aversion to water in a human subject, post-mortem examination showed the existence of a cysticercus in the brain, so that the value of this symptom as indicative of the human disease was lessened; it is also noteworthy that there was no suspicion that the individual had been bitten by a rabid animal. It will, of course, be conceded, however, that the presence of the parasite did not preclude a possibility that the patient was suffering from a bite, for the two diseases may have coexisted.

Incidentally it may be mentioned here, that spasms of the pharynx and laynx are now very universally held to be the most characteristic phenomena, and should be diligently sought for in a suspected case. At all events, it is plain that we are not lacking in good reasons for dropping the word hydrophobia from a scientific nomenclature. This view was taken some years ago by Fleming in his classical work;* though, for practical reasons, he did not venture to propose too radical a change. He suggested, however, that the old and and well-known name Rabies should be applied to the non-human form, and recent veterinary writers, especially on canine matters, have followed out this proposal. Madness, to use the English synonym, marks very clearly certain symptoms that accord with the actions and appearance of the affected animals, but has no appropriateness when the victim is a human being, for there are no psychical symptoms that convey the idea of madness in our everyday use of the word. And yet though we recognize that rabies is not an appropriate name for the human disease, it seems a little remarkable that we are

---

* The *Lancet*, Feb. 14, 1880, "Rabies and Hydrophobia." London: 1872.

not ready at once witn a new one, that is fitted to describe the clinical phenomena, or the morbid changes. Unfortunately, it must be acknowledged that our ignorance on these points is still a bar to the selection of a new name, that would have scientific value or give any general satisfaction. Perhaps it is better, as Fleming urges, to adhere for the present to the word hydrophobia, which is well understood by the laity—a point of some importance in view of the public announcements that are made from time to time by the police or sanitary authorities when an epidemic is threatened. In most country districts the prophylaxis of rabies rests chiefly with thelaity, and it is therefore very desirable that the official publication of a public danger should be made as intelligible as possible. This object would be defeated by using any new word.

Can the disease originate spontaneously is a question that still appears to have an affirmative side in the minds of some writers, although most of them sustain the opposite view. The negative side is represented by Blaine, Youatt and Meynell, while confronting them are Fleming, Sir Joseph Fahrer, Tardieu and Bouley, who have stated that further inquiry is needed before a positive opinion can be given.

Fleming has distinctly affirmed that the possibility of a spontaneous origin is unassailable, adducing in support of his opinion the subjoined reasons given by Roucher, viz.: "The marked intermission in its manifestations at different geographical centres; its regular propagation from each centre; the multiplicity of cases at certain epochs of very short duration, contrasting strikingly with their rarity at other times; the intervals between the outbreaks, which are longer than the period of incubation of the virus; the small number of cases compared with the crowds of wandering dogs; the difficulty of explaining, by contagion alone, the maintenance of the disease in every region after long interruptions."

Naturally these arguments appear insignificant to the contagionists who are now in the great majority. Many of the reasons would apply equally well to the spread of small pox or syphilis. Though every contagious disease must, of course, have had its origin *de novo*, at some time or other, no thoughtful person at this day would care to assert that they are in the habit of developing spontaneously at the present time.

Taking up more important statements, the intermissions in the disease may be explained by the fact that epidemics are always more or less self-limited, even if, as in many cases, the wholesale killing of infected dogs, and the strict surveillance of all others did not stamp out the embers of the contagion. Then, again, the intermissions may have been only apparent, since " dumb " or " sullen rabies " sometimes exists where it is never suspected. A well-known instance is found in the literature, where a pet though rabid dog was nursed until death, in the owner's lap. Finally, the duration of incubation is very variable and may, in exceptional instances, last several months and even longer, probably long enough to fill up the apparent intermissions between the outbreaks.

It has long been known that horses, cattle, sheep, goats, swine, cats, domestic fowls, and wild animals, such as the wolf, fox, hyæna, etc., have rabies, but there are comparatively few instances on record of the disease in skunks, so that the following incident has some interest, certainly to American readers.

Dr. Goddard, of Texas, was called to see a little girl, eight years of age, who, while sleeping, was bitten by a rabid skunk, on the upper lid of the right eye and corresponding side of the face. The animal was soon shot, and immediately after gave forth its peculiar odor. This incidental fact is thought to be very noteworthy, because it has been claimed that a rabid skunk never emits any odor. Nine days after the bite symptoms of hydrophobia showed themselves, and death ensued in the course of time.*

Accurate statistical information has great value, and we are constantly indebted to the continental authorities for valuable data from which practical deductions can be drawn. According to F. Muller,† who furnishes the returns of the last epidemic at

* Med. Rec., vol. 16, 1879, p. 152.

† Bollinger im Ziemssen's Handbuch, III., Bd., 2 Aufl, 1876.

Vienna (from the end of 1873 to the autumn of 1875), there was a total of 332 cases of the disease, the greatest number occurring in the winter season, and the least in the warm weather. This report should have the effect of demonstrating that the season known as the " dog days" may be the one in which the disease is least rife, and especial and extraordinary precautions at that period are not indicated. The nature of the disease poison, and the special way in which it effects an entrance into the individual, are matters that seem to be surrounded with much the same uncertainty as in many other epidemic affections. Fleming* believes that the poison is organic, though he does not commit himself on the question of its fixed or volatile nature. The ingestion of meat from a rabid animal is possibly dangerous; at any rate he sides with Chauveau in believing that the virus of a contagious disorder may be absorbed through the digestive organs. There is no certainty as to the harmlessness or harmfulness of cooked meat from a rabid animal, but the disease appears to have been communicated by applying the flesh and blood of a rabid animal to the wounds of a healthy one. The milk also may be a medium, as indicated by the following incident:

A bitch that had contracted hydrophobia, continued nursing her three puppies, even when the symptoms were very evident. After the lapse of some days, two of the puppies died of hydrophobia, and five weeks later, the third puppy. Bollinger† does not believe that the virus is transported in the ordinary sense, *i. e.*, by an intermediate object. In this connection he mentions the story of a girl who pricked herself with a needle in mending a cloak that had been torn by a rabid dog. The girl is said to have died two months afterwards of hydrophobia, but the authenticity of the anecdote is doubted.

Among the methods that have been proposed to determine whether the disease is real or not, is that by inoculation. According to Galtier,‡ hydrophobia in the dog is communicable to the rabbit, and from the rabbit to others of its own species, the

* Loc.-Cit.

† Mittheil. aus d. thierærzl Praxis in Preuss. staate 21 Jahrg. S. 90.

‡ Comptes Rendus, 25 Août, 1879, p. 444.

predominating symptoms in these cases being paralysis and convulsions. The animal may live from a few hours to three or even four days after the disease has become pronounced. The period of incubation is shorter in them than in other animals, averaging only eighteen days, as shown by a study of twenty-five cases. In them the saliva of a rabid dog taken from the living animal and preserved in water retains its virulence for twenty-four hours. This last fact, it is urged, has important bearings in the matter of post-mortem examinations, where due caution should be exercised by the operators and those in attendance. The utensils used by the animal prior to death should either be sedulously avoided or scrupulously cleansed. It will be noticed that if these statements receive proper substantiation they will be strong proof against the statements of Bollinger that the disease cannot be communicated mediately, *i. e.*, through an intermediate object. The possibility of hydrophobia being produced by the bite of a dog in the incubation stage, is a matter that still invites attention. Bollinger has not been able to collect any evidence on this subject, but if, he claims, it has been proved of another inoculable disease—variola—that it may be communicated during the period of incubation,* there is no reason to doubt that infection may take place similarly in hydrophobia. From this standpoint one of the most obscure topics in the etiology of human and animal rabies is thought to be rendered comprehensible.

According to recent official returns in Prussia, eighty-one cases are given where the period of incubation in the dog was definitely obtained. The results are as follows: In 59, or about 73 per cent., the duration was from three days to six weeks. In 22, or about 27 per cent., it was more than six weeks. Of these latter, four had an incubative period, varying between seven and eight weeks; eight between nine and ten; seven between ten and fifteen; two, three and a-half months; one, the extraordinary period of one hundred and forty-three days.†

Haubner, quoted by Romberg states that in the canine

---

* See Vol. II. of Ziemssen's Cyclopædia (Am. ed.), p. 35.

† Mittheil. aus der thieraozl. Praxis im Preuss. Staate, 21 Johrg, S. 100, 1874. Ziemssen's Handbuch, III., Bd., 2 Aufl, 1876.

species 83 per cent. were found to have an incubative period which was less than two months, the longest period being fourteen months.* He puts the average at three months. In the cat, however, the interval is from two to four weeks; in the horse from fifteen days to two months; in the ox from nine days to several months. In man he places it between fifteen days and nine months.

The following case by Oemler and Günther is given to illustrate the savage disposition occasionally shown in the furious form of canine rabies and at the same time the marked inclinations to scour wildly about the country, inflicting terrible injuries far and wide.

In December of 1871, a butcher's dog began to show signs of the disease, and was particularly disposed to bite at one of its companions; he was accordingly shut up in a stall, where he fell upon a goat and two geese, tearing them to pieces. Finally after gnawing away at the door of his enclosure he managed to effect his escape. Before day break he had bitten a number of other dogs in the vicinity and then he began a tour of destruction in the neighboring villages. Finally he was shot.†

During the thirty hours of his liberty it was computed that he ran about sixty-five English miles. He had bitten dogs in all directions and even hunted them up in their kennels, visiting farm-yard after farm-yard, walls and fences being no obstacles in his way. This terrible mania for biting appears to have created general alarm among the dogs in all the villages, which is in accordance with the common impression that a healthy dog will recognize a rabid one at a considerable distance and make efforts to get away from him.

Among the attacked were nine persons coming from church. One woman was so badly injured that she had to be driven home. In all, fifteen persons were bitten, mostly in the face and on the head; of these no less than eleven died of hydrophobia. The following case of "dumb rabies" shows the difficulty of detecting it.

---

* Diseases of the nervous system, Vol. II. p. 151.

† Bollinger (Loc. Cit.)

In July, 1874, four persons in the Rhenish Palatinate* were bitten by a dog that had broken loose from his chain, and awakened suspicion by ranging wildly round the country, even forcing his way into houses. The animal was killed.

Two days subsequently he was examined by two veterinary surgeons, and the report they gave was negative, in fact they declared him entirely free from the disease or any suspicion of it. Some weeks after, three of the persons who had been bitten (one man and two boys) died of well-marked hydrophobia. The fourth, a woman, thanks to her substantial clothing, received only a contused wound and survived.

Sullen or "dumb" rabies differs from the previous form in that there is paralysis of the lower jaw. About fifteen to twenty per cent. of rabid dogs are said to have this variety. The dog is described by Fleming as lying rolled up. He gradually wastes away and dies about fifteen days after the first symptoms have shown themselves.

* Bollinger, (Loc. Cit.)

BIBLIOGRAPHY.

FLEMING.—Rabies and Hydrophobia. London, 1872.

BOLLINGER.—Ziemssen's Handbuch. Band. III. 2 Aufl, 1876.

FAHRER, Joseph.—Brain. Vol. I, 1879.

GALTIER.—Comptes Rendus, 25 Août, 1879.

HART.—Prelim. Report on Rabies and Hydrophobia. Brit. Med. Jl., Aug. 17, 1878.

Mittheil aus der Thieraerztl, Praxis in Preuss-Staate, 21. Jehrgang, S. 100, 1874.

FRIEDREICH.—Deutsch, Arch. f. Klin. Med. xxiv., 1879.

KOWELEWSKY, P.—Memorabilien xxv., 3, 1880.

MOREAU.—Veterinary Jl. Dec., 1877.

FOOTE.—Dublin Jl. of Med. Sci. Oct., 1879.

KOLESMIKOFF.—Centralbl. f. d. Med. Wiss. 1875, S., 853.

BENEDIKT.—Virchow's Archiv. Band, lxiv. Hft. 4, S., 557.

FEREOL.—Case of Rabid Hydrophobia. (Translated by Dr. B. Ellis), Ohio Med. and Surgical Jl., 1877.

PAUL, J. and C.—Notes on Hydrophobia. (Translated by Dr. B. Ellis), Ohio Med. and Surgical Jl., 1877.

BOULEY.—Ann. d'Hygeine Publique 2, 1879.

HAMMOND.—Dis. of the Nervous System. 1879.

FITZ AND SHATTUCK.—Bost. Med. and Surgical Jl., August 29, 1878.

LINDEMANN.—Berl. Klin. Woch, 4, 1879

Lancet, Oct 18, 1879.

SOUTHAM.—Lancet, Sept. 6, 1879.

ROMBERG.—Dis of the Nervous System Vol. II.

GODDARD.—Med. Rec. Vol. 16, 1879, p. 152.

TROUSSEAU.—Clin. Med. Vol. I.

HAGGART.—St. Louis Clin. Review. March 15, 1880.

Lancet, Feb. 14 1880; Oct 18, 1879

Med. Record, 1877, p. 472; Aug. 9, 1879.

GOWERS.—Trans. of the Path. Soc. London, 1877.

CURTIS, T. B.—Boston Med. and Surg Jl., Nov. 7, 1878.

